# Response of leopard geckos (*Eublepharis macularius*) towards a multimodal cue simulating a predator

**DOI:** 10.1093/beheco/arag062

**Published:** 2026-06-05

**Authors:** Petra Frýdlová, David Hirschler, Aleksandra Chomik, Eliška Pšeničková, Eva Landová, Daniel Frynta

**Affiliations:** Department of Zoology, Faculty of Science, Charles University, Prague 2, CZ-128 44, Czech Republic; Department of Zoology, Faculty of Science, Charles University, Prague 2, CZ-128 44, Czech Republic; Department of Zoology, Faculty of Science, Charles University, Prague 2, CZ-128 44, Czech Republic; Department of Zoology, Faculty of Science, Charles University, Prague 2, CZ-128 44, Czech Republic; Department of Zoology, Faculty of Science, Charles University, Prague 2, CZ-128 44, Czech Republic; Department of Zoology, Faculty of Science, Charles University, Prague 2, CZ-128 44, Czech Republic

**Keywords:** Gekkota, kairomones, predator-prey interactions, risk assessment, sensory ecology, snake, vomerolfaction

## Abstract

Predator detection is a fundamental survival mechanism that has shaped the evolution of diverse antipredator strategies across animal taxa. The capacity to detect predators depends on the sensory modalities animals rely upon, and the integration of multiple sensory channels—multimodal perception—may enhance detection efficiency and improve survival. However, the functional significance and relative importance of multimodal perception in reptiles remain insufficiently understood. In this study, we experimentally disentangled the effects of individual sensory modalities—chemical, visual, and mechanosensory—by testing them independently and in combination, thereby assessing their relative and synergistic contributions to antipredator behavior in leopard geckos. Our results show that chemosensory information acts as the primary driver for both sensory assessment and active defense. While visual and mechanosensory stimuli did not independently increase tongue-flicking frequency compared with the control, they functioned as modulatory cues that significantly amplified chemical sampling in a multimodal context. However, overt antipredator behaviors, such as defensive posturing and mouth-open displays, were triggered exclusively by chemosensory stimuli, highlighting the dominant role of chemical cues in predator detection. Our statistical modeling suggests that sensory integration predominantly follows an additive pattern, where secondary modalities heighten alertness and facilitate threat assessment, but the initiation of costly defensive action remains strictly gated by chemoreception. These findings underscore the dominance of chemical cues for predator recognition in nocturnal environments, where multimodal inputs amplify attention but are not essential for eliciting a full defensive response.

## Teaser

How do leopard geckos detect a predator in the dark? We found that while vision and vibrations increase their alertness, geckos won’t actually attack or flee until they smell the threat. Relying on multiple senses helps geckos stay vigilant but scent is the final trigger for action. This smell-first strategy prevents them from wasting energy on false alarms, illustrating how nocturnal animals prioritize certain signals to survive.

## Introduction

Because the outcome of a predator-prey interaction can be fatal for the prey (Blanchard et al. 1990), natural selection on antipredator behaviors is expected to be particularly strong (Brodie and Brodie 1999). As a result, prompt predator detection and effective deterrence have evolved as key adaptive responses to predation pressure. Nevertheless, antipredator behavior is also associated with significant costs, as it requires animals to expend energy (eg, during escape or active defense) or reduce their activity, thereby losing opportunities for other fitness-related activities, such as foraging or mating (Labra and Niemeyer 2004; Farallo et al. 2010). These costs may be particularly pronounced in taxa facing strict energetic constraints, such as ectothermic reptiles, in which antipredator responses can directly conflict with limited basking opportunities (Cooper 2000). Thus, antipredator behavior reflects a cost-benefit trade-off: responses should occur only when the expected benefits outweigh the costs, and rapid detection of predators allows individuals to avoid energetically costly or dangerous confrontations (Stankowich and Blumstein 2005; Kelley and Magurran 2006; Pafilis et al. 2009; Samia et al. 2016).

Effective predator avoidance requires that prey accurately recognize predators and assess the degree of risk they represent. The sensory modalities available to an organism strongly constrain how this information is detected and processed. In many species, vision serves as the primary sense for detecting predators (Cooper 2008; Pessoa et al. 2014). However, visual cues are not always reliable or accessible. Predators may remain concealed, lying in ambush, or the environment may limit visibility, making visual recognition difficult or even impossible. In such cases, animals must rely on alternative sensory channels. One commonly used alternative is chemoreception; chemical cues can indicate the nearby presence of a predator, although typically without revealing its precise location (Hartman and Abrahams 2000; Amo et al. 2004; Hickman et al. 2004). Furthermore, mechanosensory cues, such as substrate-borne vibrations, can provide critical information about a predator's movement and proximity, even in complete darkness or dense cover (Young 2003; Jung et al. 2020). Similarly, acoustic signals allow for long-range detection and can alert prey to predators that are not yet within the visual field (Vitousek et al. 2007; Ito and Mori 2010; Magrath et al. 2015).

Given the complexity of natural environments, combining multiple sensory modalities likely enhances predator avoidance, thereby increasing survival and reproductive success (Hartman and Abrahams 2000; Amo et al. 2006). In their influential paper, Partan and Marler (1999) classified multimodal signal components as either redundant or nonredundant. Redundant components elicit similar responses when presented alone and serve as backup signals, ensuring information transmission in noisy environments (Krebs and Dawkins 1984). In contrast, nonredundant components convey different information and may increase informational richness per unit time (Møller and Pomiankowski 1993). When combined, redundant components can produce equivalent responses or, more commonly, enhance the receiver's reaction (Partan and Marler 1999). Nonredundant components may act independently, interact asymmetrically (eg, dominance or modulation), or together elicit novel responses (Partan and Marler 1999). While this classification was originally developed for active signaling, it provides a robust framework for understanding how prey integrates passive multimodal cues. Unlike signals evolved for conspicuousness, predator-derived cues, such as metabolic odors or movement noise, are unintentional by-products. Since natural selection favors predators that minimize such detectable traces, individual cues are often faint or ambiguous.

In this context, the framework of Partan and Marler (1999) was formally extended to predator risk assessment by Munoz and Blumstein (Munoz and Blumstein 2012), who highlighted how multimodal perception is essential for reducing the impact of environmental uncertainty. Such uncertainty inevitably generates trade-offs, as prey risk either missing real threats if cues are underestimated or wasting time and energy if they are overestimated (Sih 1986; Caro 2005; Bradbury and Vehrencamp 2011; Brown and Godin 2023). By combining various sensory signals, prey can better detect even stealthy predators and improve the accuracy of their defensive decisions.

Despite the theoretical importance of this integration, empirical research has remained somewhat narrow in scope, especially in squamate reptiles. Although several studies have explored multimodal predator detection, they have traditionally focused on bimodal interactions, primarily along the vision-chemoreception (Amo et al. 2004; Cliff et al. 2022) or visual-acoustic axes (Huang et al. 2011; Elmasri et al. 2012). In particular, research integrating mechanosensory systems into a broader multimodal framework is almost entirely lacking, and the functional significance of higher-order integration—combining more than 2 modalities—remains poorly understood. Moreover, a critical unresolved question is whether the combination of multiple predator cues leads to a synergistic enhancement of the response (where the predator is perceived as more threatening than the sum of its parts) or follows a purely additive rule of integration. Addressing this requires not only a trimodal framework but also a strictly controlled experimental design that isolates individual cues to prevent confounding effects. Our study addresses these gaps by examining a trimodal sensory context (visual, chemical, and mechanosensory). By testing observed responses against additive null models, we provide a more ecologically realistic representation of how prey navigates complex and dangerous landscapes.

Testing these theoretical predictions regarding cue integration requires a model organism with a well-balanced sensory repertoire. The leopard gecko (*Eublepharis macularius*) provides an ideal system for this purpose, as its crepuscular ecology necessitates a sophisticated integration of visual, chemical, and mechanosensory systems. Beyond its sensory suitability, this species is a well-established laboratory model extensively utilized across diverse fields, including sensory biology (Cooper and Steele 1997; Russell et al. 2014; Higham and Schmitz 2019), hybridization (Jančúchová-Lásková et al. 2015), body growth (Frynta et al. 2018; Frýdlová et al. 2020), physiology and brain function (Crews et al. 1997; Sakata et al. 2000), as well as spatial navigation and memory (Landová et al. 2023, 2025; Chomik et al. 2026). Moreover, the development of antipredator behavior (Landová et al. 2013) and responses to live snakes have been studied in detail (Landová et al. 2016). Juvenile and adult geckos display specific antipredator responses (avoidance, vocalization, escape, and biting) when exposed to nonspecific aversive stimuli, such as water spraying or gentle stick poking at the base of the tail (Landová et al. 2013), which simulate a predator attack. A broad repertoire of antipredator behaviors, including high and low postures, tail-waving, and freezing, has been documented in response to a live snake (Landová et al. 2016). Furthermore, leopard geckos exhibit comparable antipredator behavior in response to chemical cues from snakes, such as shed snakeskin (Landová et al. 2021). A key advantage of this recently developed bioassay lies in its methodological simplicity, as it enables the isolated presentation of a predator's chemical cue without the confounding involvement of other sensory modalities. When combined with the leopard gecko's suitability as a laboratory model—due to its established research history and ease of maintenance—this system allows for a robust evaluation of how multimodal cues are prioritized and integrated.

In the present study, we aim to experimentally disentangle the effects of individual sensory modalities—chemical, visual, and mechanosensory—by testing them independently as well as in combination, thereby assessing their relative and synergistic contributions to antipredator behavior. The specific aims of this study are: (I) to compare antipredator reactions in leopard geckos exposed to stimuli engaging either single or multiple sensory modalities; and (II) to assess the relative dominance of individual modalities in predator detection by experimentally isolating their effects. We specifically predict that multimodal cues would elicit more robust defensive responses than any unimodal stimulus due to increased detection reliability. Furthermore, as a crepuscular squamate, we predict that the leopard gecko would exhibit a strong reliance on vomerolfaction as its primary modality for predator identification, with mechanosensory cues acting as supplementary triggers that enhance the intensity of the response.

## Materials and methods

### Experimental animals

Experimental animals were adult leopard geckos (*Eublepharis macularius*; Blyth, 1854), a species from the family Eublepharidae, Gekkota. They represent the third filial generation descended from a wild-caught parental generation from Pakistan (Jančúchová-Lásková et al. 2015; Frynta et al. 2018). The founding parental generation (F_0_) consisted of 48 individuals. This was subsequently bred to approximately 400 individuals (F_1_, F_2_), ensuring sufficient genetic variability in our experimental population (F_3_). We tested a single cohort of 42 age-matched individuals (9 males and 33 females), which were sexually mature and 4 to 5 years old. Due to logistical reasons (parallel testing), the sample size was slightly smaller for the control (39) and triple-modality tests (*N* = 40) than for the live-snake experiment (*N* = 42). All eggs were incubated at 28.5 ± 0.5 °C to ensure standardized developmental conditions, as incubation temperature in this species is known to influence both sex determination (Viets et al. 1993) and subsequent behavior (Flores et al. 1994; Sakata and Crews 2003). Moreover, this temperature is preferred by females themselves (Bragg et al. 2000). Although this incubation regime results in a female-biased sex ratio, it is unlikely to confound our results. Our previous large-scale research involving 585 individuals has demonstrated that sex does not significantly affect antipredator behavior in this species (Landová et al. 2016), thus justifying the inclusion of both sexes in the analysis.

### Lizard husbandry

Experimental animals were housed in 1 room with a 12 h/12 h light cycle. Lights turned on and off gradually to simulate the rising and setting of the sun. The room temperature ranged from 26 to 28 °C, the preferred temperature range of wild geckos (Bergmann and Irschick 2006). Hotter basking sites were available on heating cables. Geckos were kept individually in glass terraria measuring 70 × 30 × 30 cm (L × W × H). Animals were allowed access to water with vitamins (Kombisol AD_3_E, Trouw Nutrition Biofaktory) ad libitum, and were fed with mealworms (*Tenebrio molitor*) dusted with vitamins and minerals (Roboran D, UniVIT) weekly. Body weight was measured to the nearest 0.01 g using a digital microbalance (MH-200 series) upon the completion of each major testing phase. Sex was determined by the presence or absence of pre-anal pores and hemipenal swelling and was included in the analysis as a binary factor (0 = females, 1 = males). These variables were used as covariates to account for potential effects of sex-specific strategies and differences in body condition on behavioral responses.

### Experimental design and set-up

All experiments took place in spring 2023 in home cages to avoid confounding factors such as human interference and novel environments (Grieco et al. 2021). Two days before testing, 30 × 30 cm perforated polystyrene partitions were added to each cage to reduce the space available to geckos to the front 30 × 30 × 30 cm portion, and all hides (tree bark, boxes) were made inaccessible. This ensured that geckos were accessible during testing and sufficiently well-lit for accurate evaluation of displayed behaviors. Although hides were removed to ensure unobstructed observation, geckos had a 48-h acclimation period to the modified space, where full thermoregulation remained possible via heating cables. No signs of stress were observed during this period, as the animals maintained their typical sedentary behavior. Simultaneously, magnets, which are used to secure the door of each terrarium, were dampened by the application of several layers of aluminum foil. This was done to reduce vibrations caused by opening terraria, which were previously suspected to cause increased reactivity in leopard geckos (Landová et al. 2021).

### Stimuli preparation

To elicit antipredator reactions, we used snake exuviae (shed snakeskin), which were collected from adult diadem snakes (*Spalerosophis diadema*, Schlegel, 1837) within 24 hours after being shed and stored in a sealed glass container at temperatures of −20 °C for a maximum of 2 months. The diadem snake is an allopatric snake belonging to the family Colubridae, which changes its activity period according to the season. It is diurnal during the winter, autumn, and spring, but becomes nocturnal and crepuscular during the summer (Chippaux and Jackson 2019). This species and also the smell of the exuviae have been shown to elicit significant antipredator responses in subadult (Landová et al. 2016) and adult (Landová et al. 2021) leopard geckos. As a control stimulus, we utilized pieces of transparent HDPE (high-density polyethylene). HDPE lacks detectable odor and has been previously validated as a nonreactive stimulus for this species (Landová et al. 2021). Exuviae were stored separately and handled with separate tools in order to avoid contamination. Both were cut into pieces (approximately 1 × 1 cm) and presented using either metal tweezers (approximately 10 cm between the researcher's hand and the presented stimulus, experimenter 30 cm from the gecko) or a telescopic fishing rod (approximately 40 cm from hand to stimulus, experimenter 100 cm from the gecko). We affixed a piece of exuvia or control (HDPE) to the tip using paperclips. The basic procedure for all tests involved presenting either a piece of HDPE or a chemical stimulus in front of the gecko's snout. To ensure detection of nonvolatile chemical cues requiring vomeronasal analysis, stimuli were presented approximately 3 mm from the snout, allowing the animals to perceive chemical cues through tongue-flicking or direct contact. This proximity did not induce startle reactions, as confirmed by our previous validation (Landová et al. 2021) and the lack of response to the neutral stimulus in the Control test (see below). The stimulus was presented only when the gecko faced out of the home cage towards the experimenter, thereby effectively randomizing the direction of presentation and minimizing potential lateralization effects.

### Control test

To prove the responsiveness of our experimental animals towards the examined stimuli, we conducted a control test adopted from our previous study (Landová et al. 2021). This included 39 adult leopard geckos, each of which was subjected to a sequence of 3 treatments in their home terraria. Geckos were predator-naïve and had no prior experience with snake predators, snake exuvia, or tests of this nature. First, a piece of HDPE was presented to the tip of the snout using metal tweezers (Control 1). Subsequent behavior was recorded for 90 s. Secondly, a piece of exuvia was presented in the same manner while recording for 90 s. The third treatment was identical to the first (Control 2). Between each treatment, geckos were given an approximately 60-minute rest period. This established protocol (Landová et al. 2021) was used to assess the novelty effect of presenting a new object (Control 1) and potential sensitization or recovery following exposure to the predator cue (Control 2).

### Triple modality test procedure

**Figure 1:**
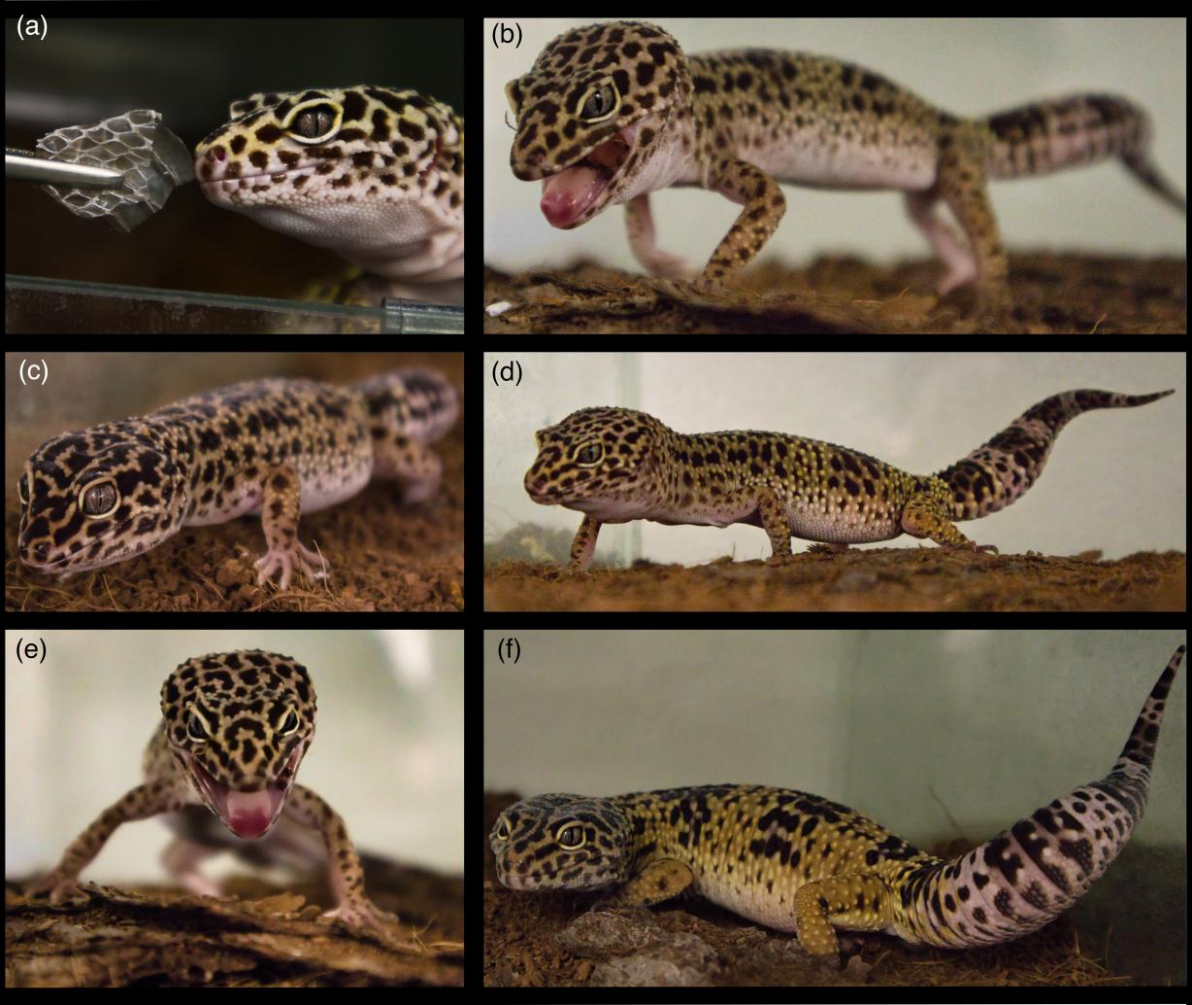
The selected behavioral elements exhibited by leopard geckos towards the exuvia (shed snakeskin). a) Confrontation with the exuvia, b) high posture with mouth-opening threat from lateral view, c) low posture, d) high posture with tail-waving, e) high posture with mouth-opening threat from frontal view, f) low posture with tail-waving. Photos by David Hirschler.

We tested 40 individuals in this test. The main test consisted of 8 different treatments designed to isolate the effect of individual cues and compare their significance when presented individually or in combination. Treatments were presented in a pseudo-randomized order. Tests were conducted over 8 days to ensure that each animal was only tested once per day. Treatments were designed to present a combination of stimuli targeting 3 different sensory modalities: chemical, visual, and mechanosensory, as well as control stimuli, meant to elicit no antipredator reaction. The presented chemical stimulus was in the form of a 1 × 1 cm piece of exuvia presented to the tip of the snout (Fig. 1a). As a chemically neutral control, we used a 1 × 1 cm piece of HDPE. Visual cues were determined by the presentation method. When presented using metal tweezers (12 cm long), we considered the researcher's hand (in gloves) with tweezers as a visual stimulus (a large moving object placed near the gecko). As a visual control, meant to minimize the effect of visual stimulation, we presented the stimulus using a thin telescopic fishing rod, allowing the researcher to present chemical cues without placing their hand near the animal subject. To minimize variability, all stimuli were presented by a single researcher (D.H.) following a standardized protocol for movement speed and distance. The hand-held tweezers and the thin rod were used to represent complex and minimized visual cues, respectively. Although the lack of response to the hand-held HDPE control shows that the researcher's hand was not perceived as a primary threat, this design allowed us to test whether the presence of a larger visual object acts as a secondary cue that modulates the response to the predator's scent (ie, multimodal integration). Mechanosensory stimulation was achieved by firmly tapping the terrarium glass 4 times in a single continuous sequence with metal tweezers immediately before presenting chemical cues. The resulting sound reached an intensity of approximately 80 dB (measured at the position of the gecko's head). This intensity is well above the auditory threshold of the leopard gecko, which exhibits peak sensitivity to frequencies between 500 and 2,000 Hz at significantly lower intensities (Werner and Wever 1972). In addition to the auditory component, the tapping caused substrate vibrations. Together, these stimuli were intended to serve as an alert, potentially increasing the geckos’ reactivity to subsequent cues. As a control for this stimulus, we avoided tapping and ensured the terraria magnetic mechanisms were padded.

Treatment 1 (T1) consisted of a piece of HDPE presented using a telescopic rod without a mechanosensory alert, which represents a control test with no involved modality. Treatment 2 (T2) consisted of a piece of HDPE presented using a telescopic rod with a mechanosensory alert (mechanosensory modality). Treatment 3 (T3) consisted of a piece of HDPE presented using a pair of metal tweezers without a mechanosensory alert (visual modality). Treatment 4 (T4) was represented by a piece of exuvia presented using a telescopic rod without a mechanosensory alert (chemical modality). Treatment 5 (T5) consisted of a piece of HDPE presented using a pair of metal tweezers with a mechanosensory alert (visual and mechanosensory modality). Treatment 6 (T6) was represented by a piece of exuvia presented using a telescopic rod with a mechanosensory alert (chemical and mechanosensory modality). Treatment 7 (T7) contained a piece of exuvia presented using a pair of metal tweezers without a mechanosensory alert (chemical and visual modality). Treatment 8 (T8) consisted of a piece of exuvia presented using a pair of metal tweezers with a mechanosensory alert (chemical, visual, and mechanosensory modality). Stimuli were presented to geckos, and reactions were recorded for 90 s using a visual spectrum mirrorless camera (Canon EOS M50) for further analysis of displayed behaviors.

### Multimodality test procedure with a live snake

In this test, we used a live snake (*Spalerosophis diadema*) as a visual stimulus because we had doubts about the relevance of the visual stimulus from the previous Triple modality test. The mechanosensory component was omitted here because it showed only a weak effect in the previous tests. We tested 42 leopard geckos in only 2 modalities: visual and chemical. As a chemical stimulus, we once again used a 1 × 1 cm piece of exuvia from a diadem snake, with a 1 × 1 cm piece of HDPE serving as a control. Both were presented using a telescopic fishing rod to avoid the possibility of the researcher's hand affecting the gecko's behavior. During this test, we introduced a different type of visual stimulus. The visual stimulus consisted of a live adult diadem snake in a sealed glass container (17.5 × 7 × 8.5 cm; L × W × H). As a control, we used an identical empty glass container.

Each gecko went through 4 treatments in a pseudo-random order over 4 days (1 treatment per day). Treatment 1 (T1) consisted of an empty glass container being placed into one side of the terrarium and a piece of HDPE presented to the gecko's snout using a telescopic rod (no modalities involved). Treatment 2 (T2) was a live snake in a sealed glass container placed into one side of the terrarium, and a piece of HDPE presented to the gecko's snout using a telescopic rod (visual modality). Treatment 3 (T3) included an empty glass container being placed into one side of the terrarium and a piece of exuvia presented to the gecko's snout using a telescopic rod (chemical modality). Treatment 4 (T4) consisted of a live snake in a sealed glass container being placed into one side of the terrarium and a piece of exuvia presented to the gecko's snout using a telescopic rod (visual and chemical modality). The placement of the glass container was randomized (left vs. right) for each trial to avoid side bias. Stimuli were presented to geckos, and reactions were recorded for 90 s for further analysis of displayed behaviors.

### Behavioral analysis

We employed the Activities—Event Recorder version 2.1 (Vrba and Donát 1993) to evaluate behavior during experiments. We categorized and marked displayed behavior from the video footage we gathered throughout all experiments. The behavioral scoring was performed manually by a single experienced observer (D.H.). While a blind protocol was not feasible due to the nature of the experimental setup, we minimized potential bias by using clearly defined, nonambiguous behavioral categories (eg, tongue-flicking, biting). To ensure internal consistency, 15% of the videos were re-scored by the same observer after a 2-month interval, showing 100% agreement in the binary categories. We utilized the ethogram developed by Landová et al. over the course of previous years (Landová et al. 2013, 2016). We observed and marked the following types of behavior: (I) tongue flicking–number of tongue flicks directed toward presented stimuli as a measure of the gecko's interest and attention. (II) No observable response–the animal showed no change in behavior and continued with its baseline activity (eg, resting or routine locomotion) despite the presence of the stimulus. Defensive behaviors included: (III) high posture–geckos raised themselves into a high posture, extending their legs, often hunching their back (Fig. 1b–d). During this posture, the tail is usually at least partially raised; (IV) mouth-open threat–wide opening of the mouth, revealing the contrasting pink interior along with the bright red tip of the gecko's tongue (Fig. 1b and e). The mouth will usually be held open for extended periods; (V) biting–usually follows a mouth-open threat. It is a swift, painful bite utilizing the gecko's relatively strong jaws and many small but sharp conical teeth. Geckos will either attack rapidly, quickly letting go before resuming a threatening stance, or they may latch on to the target for several seconds; (VI) low posture–a low stance, pressing the body to the ground (Fig. 1c and f); (VII) tail-waving—a threatened gecko may raise its tail into the air and wave it in a slow curving motion, partially furling and unfurling repeatedly (Fig. 1b, d, f); and (VIII) avoiding–avoidance and escape behaviors from the stimulus. Avoidance behavior was scored when a hastened movement away from the presented stimulus was observed (either by simply turning the head/body, or by moving to a different part of the enclosure). To allow leopard geckos to display such behavior, the stimulus was not constantly pressed to the subjects' snouts throughout the whole 90-s duration of each test. Instead, stimuli were presented to the snout and, whenever the subject moved away from the stimulus (avoiding or ignoring it), the researcher waited in the same position for approximately 2 s before moving the stimulus to the gecko's snout again. This intermittent approach ensured continuous stimulus exposure throughout the fixed 90-s trial while providing the animal with sufficient time and space to display clear avoidance or withdrawal behaviors without being pursued. Initially, we recorded multiple behavioral parameters, including frequency, duration, and latency. However, as individuals often displayed only a subset of the total behavioral repertoire within the short 90-s trial, these continuous variables showed high zero-inflation and lacked the necessary distribution for robust statistical analysis. Furthermore, because specific defensive actions (eg, stationary high postures vs. active threat-biting) are mutually exclusive and compete for time, summing their durations or frequencies into a single metric would not yield a biologically meaningful measure. Therefore, we opted for binary scoring (1 = presence, 0 = absence), which reliably captures whether a specific antipredator strategy was triggered, regardless of its duration or frequency. This choice directly aligns with our primary biological question: testing the sensory threshold required to activate a defensive strategy, rather than evaluating the intensity or duration of the response. To further address the fact that individuals typically utilize different elements of their repertoire, we used a pooled binary composite score for the primary statistical analysis. A response was assigned if at least one active (high posture, mouth-open threat, biting, and avoidance) or passive (low posture, freezing, and tail-waving) defensive behavior was observed. This approach allowed us to represent the overall antipredator reaction more robustly than would be possible through separate analyses of individual elements, which were often characterized by low frequencies. Most behavioral elements are visible in the video recording (Videos S1 and S2 provided in Frýdlová et al. 2026).

### Statistical analysis

To visualize the overall structure of behavioral responses across different stimuli, we performed a Principal Component Analysis (PCA) as an exploratory tool to simplify the complexity in high-dimensional data. This analysis was based on the frequencies of individual behavioral elements using STATISTICA 6.0 (StatSoft, Inc. 2001). However, PC scores were not used for hypothesis testing; instead, we relied on the direct modeling of two main dependent variables: (I) the number of tongue flicks and (II) the antipredator reaction towards the stimulus (coded in a binary way). To account for the surplus of zero observations in tongue-flicking data, we used a zero-inflated negative binomial (ZINB) generalized linear mixed model (GLMM) via the glmmTMB package (Brooks et al. 2017). The binary antipredator reaction was analyzed using a GLMM with a binomial distribution and logit link function via the lme4 package (Bates et al. 2015). However, for the specific reaction to the live snake stimulus, where GLMMs produced unrealistic probability estimates, a generalized estimating equation (GEE) with a binomial distribution and logit link via the geepack package (Halekoh et al. 2006) was employed instead. The use of GEE is recommended for biological data with complex structures or when standard GLMMs fail to provide reliable estimates (Pekár and Brabec 2018). In all mixed models, treatment, body weight and sex were fixed effects, and animal identity was a random intercept. GEE models used a comparable correlation structure to account for repeated measures.

To assess the individual and combined contributions of different sensory modalities, we further analyzed the data using a factorial model. In this approach, the 8 treatments were decomposed into 3 binary fixed factors: chemical cues (present/absent), visual cues (present/absent), and mechanosensory cues (present/absent). We tested for all main effects and their 2-way and 3-way interactions. The error structure (including the random effect of individual ID) and the distributions (ZINB for tongue flicking and binomial for defensive reactions) remained identical to the primary models described above.

Estimated marginal means (EMMeans) with Tukey’s HSD adjustment were used for post hoc comparisons between treatments in all models, using the package emmeans (Lenth 2024) in R, version 4.5.2 (R Development Core Team 2026). Model validity was confirmed using the DHARMa package (Hartig 2022); visual inspection of 1,000 simulated residuals showed no significant deviations from the expected distribution, overdispersion, or heteroscedasticity (all *P* > 0.05). For visualization, we used ggplot2 (Wickham 2016). While the model estimations were performed on the link scales, for ease of interpretation, we report back-transformed EMMeans and probabilities along with their 95% confidence intervals (CI) in the text and figures. To evaluate the patterns of sensory integration within Triple modality test, we compared observed responses (EMMeans, *y*-axis) derived from tongue-flicking data against a theoretical additive null model (*x*-axis). These predicted values were calculated by summing individual marginal effects relative to the control baseline. The alignment of observed data with the 1:1 identity line was used to assess whether the responses deviate from an additive expectation. Analysis reported in this article can be reproduced using the data provided by Frýdlová et al. (2026).

## Results

### Control test

Out of the 39 trials in which a piece of exuvia was presented to the snout, 13 geckos exhibited a wide array of antipredator reactions. This sharply contrasts with only 2 antipredator reactions (high posture) observed in the 78 trials involving the control stimulus (HDPE), a difference that was statistically significant (χ^2^ = 44.61, df = 2, *P* < 0.0001). This finding suggests that HDPE serves as an appropriate control stimulus, while exuviae appear to be an effective chemical trigger for an antipredator response. Leopard geckos displayed significantly more tongue-flicks toward snakeskin (mean = 7.65, 95% CI: 5.56 to 9.74) compared with the first control (mean = 2.79, 95% CI: 1.87 to 3.7, *P* < 0.0001). The second control did not differ significantly from the first (mean = 2.3, 95% CI: 1.43 to 3.17, *P* = 0.398).

### Triple modality test

**Figure 2:**
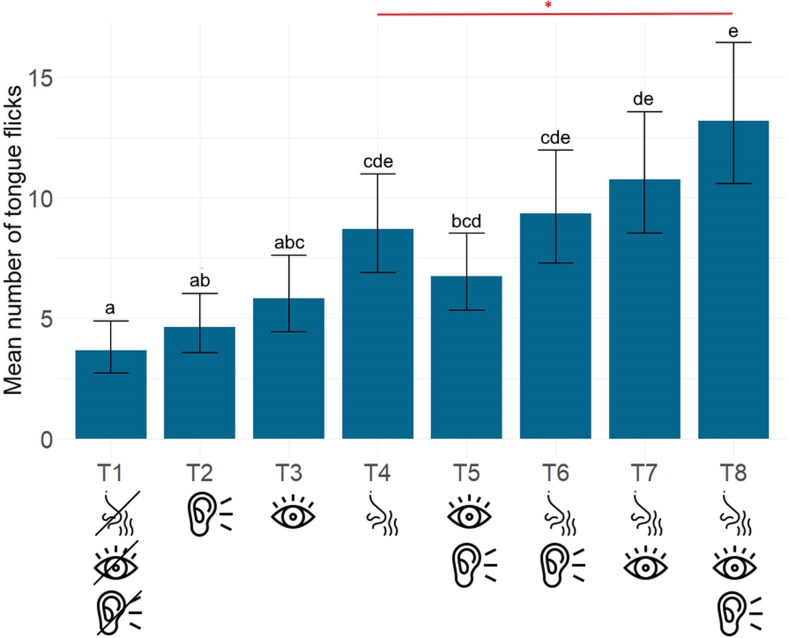
Frequency of tongue flicks exhibited by leopard geckos in the triple modality test. Data are presented as model-predicted means ± 95% CI. Letters above bars indicate significant differences based on post-hoc comparisons (Tukey HSD) for the full dataset (T1–T8); groups sharing at least one letter do not differ significantly. Significance bracket and asterisk indicate significant difference within the reduced dataset (T4, T6–T8). Significance levels: * *P* < 0.05. Treatment 8, which involved all modalities (chemical, visual, and mechanosensory), attracted the most attention, as evidenced by tongue flicking, followed by T7 (chemical and visual), T6 (chemical and mechanosensory), and T4 (chemical).

We found that a combination of more modalities attracted the most attention in the form of exploration by tongue flicks, but not increased antipredator reactions. When we explored the number of tongue flicks, we uncovered a significant effect of treatment (χ^2^ = 80.28, df = 7, *P* < 0.0001), but not body weight (χ^2^ = 0.0053, df = 1, *P* = 0.942) or sex (χ^2^ = 1.09, df = 1, *P* = 0.296). The treatments that combine 2 or 3 modalities triggered a higher number of tongue flicks compared with the control group (mean = 3.66 flicks, 95% CI: 2.73 to 4.89). Specifically, the highest mean number of tongue flicks was elicited by the combination of all 3 modalities (T8: mean = 13.18 flicks, 95% CI: 10.57 to 16.44, *P* < 0.0001). This was followed by Treatment 7 (chemical and visual: mean = 10.75 flicks, 95% CI: 8.53 to 13.55, *P* < 0.0001), Treatment 4 (chemical only: mean = 8.69 flicks, 95% CI: 6.89 to 10.97, *P*  *<* 0.0001), Treatment 6 (chemical and mechanosensory: mean = 9.34 flicks, 95% CI: 7.29 to 11.96, *P*  *<* 0.0001), and Treatment 5 (visual and mechanosensory: mean = 6.73 flicks, 95% CI: 5.32 to 8.52, *P*  *<* 0.001, Fig. 2, for contrast see Table S1 in Supplementary material).

**Figure 3:**
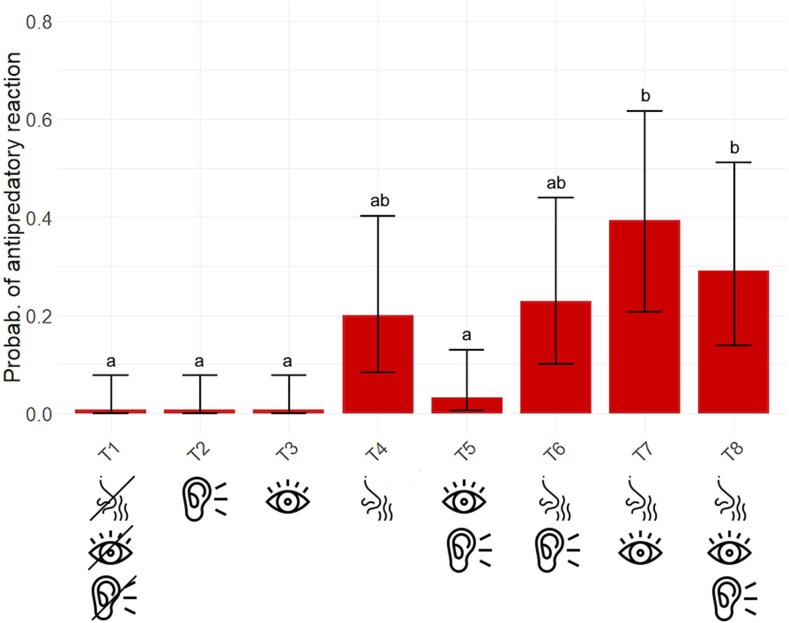
Predicted probability of antipredator reaction exhibited by leopard geckos in the triple modality test. Data are presented as model-predicted probabilities ± 95% CI derived from the binomial GLMM. Letters above bars indicate significant differences for the full dataset (*P* < 0.05, Tukey HSD adjustment); treatments sharing a letter do not differ significantly. While antipredator reactions were triggered only in treatments involving chemical cues (T4, T6, T7, T8), no significant differences were found within this reduced scent-present subset. This indicates that the presence of chemical cues is the primary driver of the response, with additional sensory modalities not significantly increasing the probability of reaction.

Regarding the antipredator reaction, we found a significant effect of treatment (χ^2^ = 34.74, *P*  *<* 0.0001) on the reaction in the Triple modality test, while body weight (χ^2^ = 2.28, *P*  *=* 0.13) and sex (χ^2^ = 0.57, *P* = 0.45) were not significant factors. Post-hoc comparisons showed that the highest probabilities of response occurred in treatments involving chemical stimuli. Specifically, responses were significantly more frequent in Treatment 7 (chemical and visual: prob = 0.40, 95% CI: 0.21 to 0.62, *P* = 0.0071) and Treatment 8 (chemical, visual and mechanosensory: prob = 0.29, 95% CI: 0.14 to 0.51, *P* = 0.0249) compared with the control. Treatment 6 (chemical and mechanosensory: prob = 0.23, 95% CI: 0.10 to 0.44, *P* = 0.0563) and Treatment 4 (only chemical: prob = 0.20, 95% CI: 0.09 to 0.40, *P* = 0.0841) showed similar positive trends, although they did not reach the strict significance threshold after post-hoc adjustment (Fig. 3). For a full overview, see Table S2 in Supplementary material and Videos S1 and S2 (Frýdlová et al. 2026) for recording of behavior.

To specifically investigate how secondary modalities modulate the response to the primary predator trigger (snake scent), we focused our subsequent analysis on the subset of trials where snake exuviae were present (T4, T6, T7, and T8). Treatments that did not elicit any defensive response in the full dataset (T1, T2, T3, and T5) were excluded to avoid excessive zero-inflation and to increase the power to detect synergistic effects. We employed the same model structure and distributions as in the full dataset analysis. We found no significant differences between these 4 treatments on the antipredator reactions (χ^2^ = 3.409, df = 3, *P* = 0.3327, Fig. 3, for contrasts see Table S3 in Supplementary material). Nevertheless, there was a significant overall effect of treatment on the number of tongue flicks (χ^2^ = 8.61, df = 3, *P* = 0.035, Fig. 2). Specifically, post-hoc comparisons revealed that the number of tongue flicks was significantly higher when all 3 modalities were involved in comparison to chemical cues alone (*P* = 0.0348; Fig. 2, for contrasts see Table S4 in Supplementary material).

**Figure 4:**
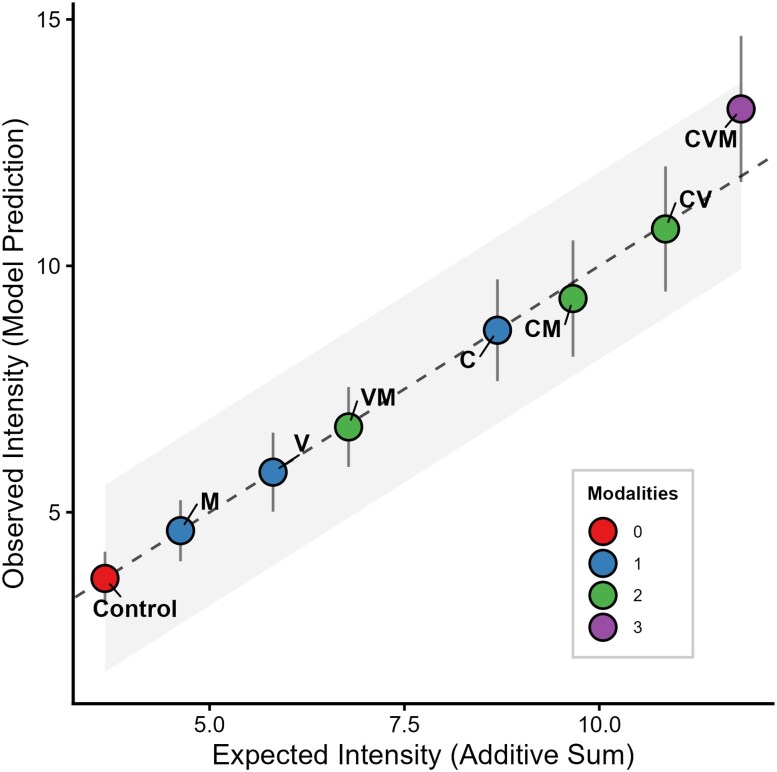
Sensory integration in tongue-flicking behavior. Observed responses (GLMM predictions) are plotted against expected values (additive sum of individual cues). The dashed line represents theoretical additivity (1:1 identity); the shaded area shows the 95% confidence zone derived from the unimodal variance. Points represent sensory combinations: Control (no stimulus), C (chemical), V (visual), M (mechanosensory), and their combinations (CV, CM, VM, CVM). The alignment of the observed data with the diagonal is consistent with an additive integration of sensory modalities, suggesting a lack of synergistic enhancement.

The number of tongue flicks was significantly influenced by chemical (χ^2^ = 62.48, df = 1, *P* < 0.0001) and visual cues (χ^2^ = 14.50, df = 1, *P* = 0.0001), while mechanosensory stimuli reached borderline significance (χ^2^ = 3.29, df = 1, *P* = 0.07). No significant interactions between modalities were detected (all *P* > 0.05). In contrast, for the probability of antipredator reactions, only the effect of chemical cues remained highly significant (χ^2^ = 8.098, *P* < 0.0044), while visual (χ^2^ < 0.01, *P* > 0.9) and mechanosensory (χ^2^ < 0.01, *P* > 0.9) cues did not show a comparable influence. Furthermore, no significant interactions between sensory modalities were detected (all *P* > 0.05). These results suggest a predominantly additive pattern of sensory integration for tongue-flicking behavior, as further supported by the close alignment of observed multimodal responses with the predicted additive null model (Fig. 4). While our data did not reveal significant synergistic enhancement, the observed patterns are consistent with the hypothesis that these cues are integrated cumulatively.

To explore the relationships between individual behavioral elements during the chemical and multimodal trials, we performed an exploratory PCA and visualized behavioral transitions. PCA, based on trials where chemical stimuli were presented, revealed that the first two factors explained 44.33% of the total variance (Figure S1 in Supplementary material). Factor 1 was characterized primarily by a gradient between defensive and exploratory behaviors; high negative loadings for biting, mouth-open threats, and high postures contrasted with tongue-flicking and cases of “no response”. This suggests a separation between active defensive strategies and exploratory or baseline activities. Factor 2 was further associated with the distinction between active and passive defensive elements. The typical progression of these behaviors followed a nonrandom sequence (Figure S2 in Supplementary material). Tongue-flicking was the most frequent initial element, typically followed by a bite or a tail wave. However, the sequence analysis also revealed that threat detection was not always preceded by visible tongue-flicking, as several reactions began directly with high postures followed by a mouth-open threat, bite, or avoidance. Passive elements, such as low postures and freezing, were rare.

### Modality test with a live snake

**Figure 5:**
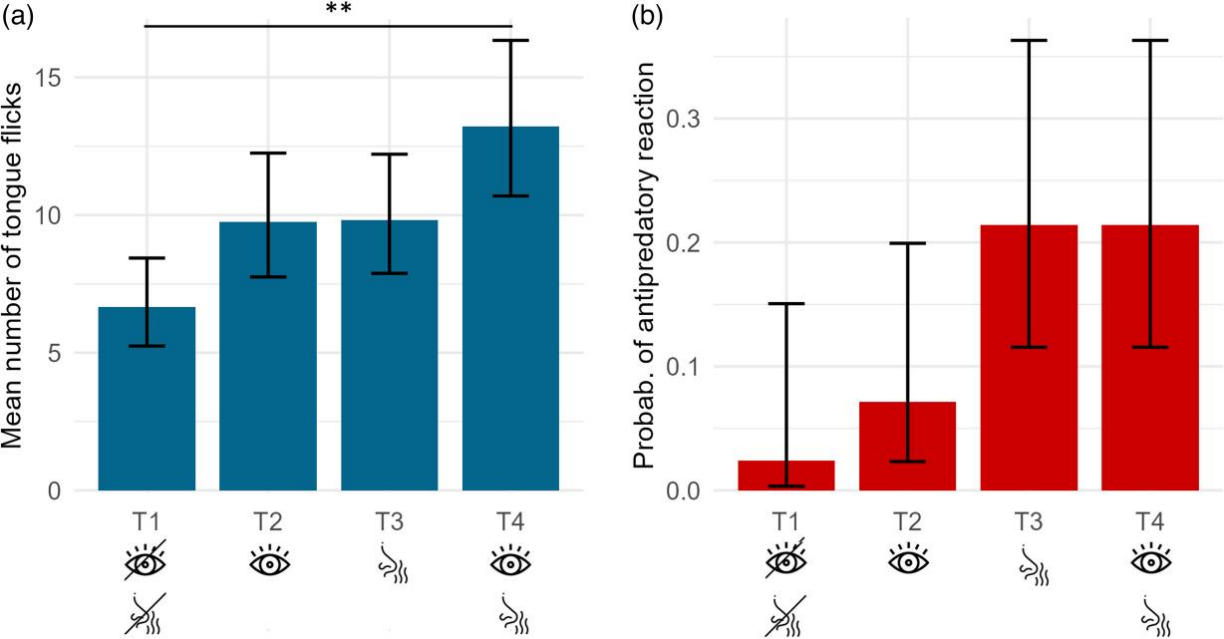
Sensory assessment and defensive responses in the Modality test with a live diadem snake. a) Mean number of tongue flicks and b) the probability of antipredator reaction exhibited by leopard geckos (model predictions from the respective GLMMs; Mean ± 95% CI). Significance levels are based on emmeans pairwise contrasts (significance levels: ** *P* < 0.01). Tongue flicking rate was significantly higher in Treatment 4 (combined chemical and visual modality) compared with the control. The probability of antipredator reaction was slightly higher if the chemical modality was stimulated (Treatment 3 and 4) by the presence of exuvia (shed snakeskin), whereas visual stimuli alone did not significantly increase defensive response compared with the control.

In the test with the live snake, the treatment was a significant factor (χ^2^ = 19.76, df = 3, *P* < 0.001) affecting the number of tongue flicks considerably. While the visual (Treatment 2) and the chemical (Treatment 3) stimuli alone did not elicit significantly more tongue flicks (Treatment 2: mean = 9.75, 95% CI: 7.75 to 12.25 and Treatment 3: mean = 9.81, 95% CI: 7.88 to 12.21, respectively) in comparison with the control (mean = 6.65, 95% CI: 5.24 to 8.44), the combination of visual and chemical modalities (Treatment 4: mean = 13.22, 95% CI: 10.70 to 16.35) triggered significantly more tongue flicks (post hoc Tukey test: *P* < 0.0001; Fig. 5a, for model contrast, see Table S5 in Supplementary material).

Conversely, we found no significant effect of the treatment on antipredator reactions (χ^2^ = 7.606, *P* = 0.055). The treatment with chemical and visual modalities (Treatment 4) elicited the same probability of antipredator reactions (prob = 0.21, 95% CI: 0.11 to 0.36) as when only the chemical modality was stimulated (prob = 0.21, 95% CI: 0.11 to 0.36). The post hoc Tukey test did not reveal a significant difference from the control treatment (prob = 0.02, 95% CI: 0.0033 to 0.15, for model contrast, see Table S6 in Supplementary material). The separate visual modality did not trigger any significant antipredator reaction (prob = 0.07, 95% CI: 0.02 to 0.20; Fig. 5b).

## Discussion

Building on our previous findings that leopard geckos react to both specific snake cues and nonspecific threats (Landová et al. 2013, 2016), this study systematically disentangles the contributions of individual sensory modalities and evaluates the role of multimodal perception in predator detection. Our results demonstrate that while leopard geckos are sensitive to various predator-related cues, chemical information acts as the indispensable primary driver for both sensory assessment and active defense. Interestingly, while visual and mechanosensory stimuli did not independently trigger an increase in tongue-flicking frequency compared with the control, they functioned as modulatory cues in multimodal contexts. When combined with chemical information, these additional inputs significantly amplified the intensity of chemical sampling (tongue-flicking), suggesting that they provide secondary confirmation that heightens the animal's alertness. In contrast, the transition to active defense followed a strict hierarchy: the probability of defensive behavior was tied exclusively to the presence of the chemical stimulus and did not increase with the addition of other modalities. This suggests a 2-stage evaluation process: while secondary modalities increase the level of alertness in the presence of a primary cue, the decision to initiate a costly, overt defensive reaction is strictly gated by chemoreception.

To further validate these findings, we conducted a follow-up test using a live snake predator while manipulating the availability of visual and chemical information. Consistent with the results from the simulated trials, sensory assessment (tongue-flicking) increased when multimodal cues were available, yet overt antipredator behaviors remained primarily tied to chemosensory input. Surprisingly, the visual presence of a live, moving predator without accompanying chemical cues elicited a defensive response only in a minority of cases. This reinforces our conclusion that for the leopard gecko, visual motion, even from a high-threat source, may serve as a trigger for increased alertness but is frequently insufficient to elicit a full defensive response unless confirmed by chemical signals. This consistent pattern across both simulated and live trials underscores the high threshold required for initiating active defense in this species.

Our findings suggest that leopard geckos primarily rely on chemical cues for predator detection, a strategy likely shaped by their nocturnal lifestyle, where visual information may be ambiguous. Despite this reliance, leopard geckos possess a sophisticated sensory repertoire. Geckos are primarily visual predators (Higham and Russell 2010) with relatively large eyes (Werner 1969) and a unique retina composed of modified cones (Walls 1944), allowing for high sensitivity across a wide range of light conditions (Loew et al. 2002; Higham and Schmitz 2019). Similarly, their skin is equipped with specialized cutaneous sensilla, particularly dense on the tail, which are tuned to detect subtle mechanical contact (Russell et al. 2014).

However, the possession of these advanced systems does not necessitate their equal involvement in triggering all behavioral stages. For an ectothermic organism, initiating an active antipredator response such as biting or posturing represents a significant energy expenditure. Our results suggest a “conservative” decision-making strategy: while highly sensitive visual and mechanosensory systems are utilized for initial sensory assessment (as evidenced by the increased tongue-flicking in multimodal trials), the threshold for costly defensive action is only reached when the threat is confirmed by the most reliable nocturnal modality—chemoreception.

In nocturnal lizards, chemical cues often provide more certain information regarding a predator's identity than visual motion (Dial and Schwenk 1996). Therefore, gating active defense behind vomerolfaction, a system specialized for detecting nonvolatile, biologically relevant compounds via tongue-flicking (Halpern and Martınez-Marcos 2003), may prevent “false alarms” and unnecessary energy loss. This explains why even the visual presence of a live predator, as seen in our follow-up experiment, was often insufficient to elicit a full defensive response without chemical confirmation. Our study thus illustrates not just a lack of response to certain stimuli, but a finely tuned sensory hierarchy that prioritizes reliability over mere detection.

This hierarchical strategy is further supported by our analysis of sensory integration patterns (Fig. 4). The observed responses align closely with the additive null model, suggesting that the combined effects of multiple stimuli follow a largely additive pattern. In this framework, the total response corresponds primarily to the sum of individual contributions without clear evidence of synergistic enhancement. Such a pattern, reflected in the clustering of data points near the 1:1 identity line, is consistent with a stable and predictable assessment process. By avoiding pronounced nonlinear amplification of threat levels, leopard geckos may maintain a balanced response that prevents excessive energy expenditure while still benefiting from the increased alertness provided by secondary sensory inputs.

Leopard geckos appear to be well adapted, from a sensory perspective, to detect and assess predation risk. The ability to detect a predator early is critical for initiating effective antipredator strategies and ultimately avoiding capture (Bateman et al. 2014). Among the primary natural predators of leopard geckos are owls and kites, as well as small mammalian carnivores, such as jackals, foxes, and mongooses (Khan 2009). Due to the speed and agility of these endothermic predators, successful escape may be limited once an attack has been initiated. In such scenarios, visual and mechanical information provide essential early warning that keeps the animal in a state of high alertness, while secondary defenses, such as antipredator vocalizations or aposematic displays, previously described in this species (Landová et al. 2013), may be deployed as a last resort.

Snakes (eg, *Ptyas mucosus, Naja naja, Eryx johnii*) also likely represent another ecologically relevant category of predators for leopard geckos (Khan 2009; Landová et al. 2016). In contrast to avian or mammalian predators, snakes often rely on stealth rather than speed, rendering early detection particularly advantageous. Our findings suggest that chemical cues serve as the primary filter for confirming a predator's identity in these contexts, providing a critical window for assessment. Behavioral defenses, such as threat posturing or the presentation of an autotomizable tail, may then serve as effective deterrents. The strong reliance on chemoreception observed in our study thus reflects an adaptation to predators that can be detected through chemical trails long before they become visually or mechanically apparent.

Previous research has demonstrated that specific antipredator responses in leopard geckos are innate and undergo significant ontogenetic shifts (Landová et al. 2013, 2016). While juvenile geckos rely more on active defensive behaviors such as vocalizations and threat-biting, adults predominantly employ escape strategies (Landová et al. 2013). These findings indicate that antipredator strategies in this species are developmentally plastic and adaptively tailored to age-specific ecological constraints and predation risk (Landová et al. 2013). While our current study focused on adults, our results complement this by showing that leopard geckos possess a portfolio of defenses (Kikuchi et al. 2023) that can be flexibly deployed. Furthermore, we have previously shown that these geckos do not display tailored responses to specific predators, but rather exhibit a generalized wariness, regardless of the predator's foraging strategy or ecological relevance (Landová et al. 2016, 2021). This consistent behavior, observed across both live snake predator presentations (Landová et al. 2016) and exposure to chemical cues, such as exuviae (Landová et al. 2021), suggests a reliance on broadly responsive strategies. Such generalized responses in other nocturnal species, including velvet geckos (Webb et al. 2009, 2010), point toward a coevolutionary trajectory where selection favors flexible response systems in species exposed to diverse and variable predator communities.

## Impact of stimulus selection

In our triple modality test, the nonspecific visual and mechanosensory stimuli elicited only a weak antipredator response. The lack of response to the visual stimulus (experimenter's hand holding tweezers) may be explained by habituation during husbandry. To overcome this, we employed a live snake as a biologically relevant stimulus in a subsequent experiment. However, even the visual presence of a moving predator, in the absence of chemical cues, elicited antipredator responses only rarely. This reinforces our conclusion that for leopard geckos, visual information primarily serves to increase alertness, potentially reflecting heightened information sampling under sensory uncertainty (Landová et al. 2021), but requires chemical confirmation to trigger active defense. However, we acknowledge that presenting the live predator behind a barrier likely constrained the ecological realism of the encounter by limiting substrate vibrations and direct interaction, which may have further attenuated the perceived threat of the visual cue. While multimodal enhancement is common in diurnal lizards (Amo et al. 2004; Cliff et al. 2022), our results suggest that in nocturnal species, the sensory hierarchy is more strictly gated by chemoreception.

An additional methodological consideration concerns the choice of mechanosensory stimulus. In our study, this was presented as a mechanical strike of tweezers against the home terrarium, producing both substrate-borne vibrations and an acoustic signal. While these stimuli triggered only occasional antipredator responses, it does not necessarily imply that this modality is unimportant in a more natural predator context. It remains an open question which type, intensity or frequency of mechanosensory input would be more suitable for reliably inducing defense in leopard geckos. It is possible that the vibrations produced by our method did not sufficiently mimic the specific frequency of a predator's movement or strike. In future research, it might be beneficial to incorporate standardized acoustic cues (eg, following Elmasri et al. 2012), more focused air-puffs simulating a predator's lunging motion, or even direct tactile stimuli in the sacral region, which we already successfully employed in our previous study (Landová et al. 2013). Moreover, the absence of a synergistic reaction may be due to stimulus incongruence. The mechanosensory, as well as visual stimulus, in the Triple modality test, was not congruent with the smell of the snake. This is supported by the increase in tongue-flicking, a behavior typical for states of uncertainty rather than a confirmed predatory context (Landová et al. 2021).

## Limitations of the study

Our study demonstrates that chemical cues are the primary driver for both sensory assessment and active defense in leopard geckos. Visual stimuli, whether a hand or a live snake, played only a modulatory role. Although a human hand is not a natural predator, periodic handling in captivity likely renders it a relevant threat. The secondary importance of visual cues is further supported by our previous findings (Landová et al. 2016), where geckos responded to a snake within their home cage but did not scale their response intensity to the snake's movement. This confirms that visual motion is insufficient to trigger or significantly amplify defense, which remains strictly gated by chemoreception. Future research should focus on congruent multimodal presentations to better simulate natural encounters, although unconstrained predator-prey studies remain limited by technical and ethical constraints.

## Conclusions

Our findings demonstrate that the chemical sense plays a central role in predator detection and the triggering of defensive behaviors in the leopard gecko. While a predator's scent alone was sufficient to elicit a distinct antipredator response, the allocation of attention, measured as tongue flicking, was significantly enhanced when multiple sensory modalities were stimulated. Statistical modeling further suggests that this sensory integration predominantly aligns with an additive pattern, where the combined effect corresponds to the sum of individual components without significant synergistic enhancement. This finding suggests a strategy that prioritizes reliability over mere detection and indicates that while chemosensory input is essential for triggering energetically expensive defensive actions, the integration of additional visual and mechanosensory cues serves to heighten vigilance and improve early threat assessment. Such a sensory hierarchy allows leopard geckos to maintain high alertness while avoiding costly false alarms in their complex, nocturnal environments.

## Supplementary Material

arag062_Supplementary_Data

## Data Availability

Analyses reported in this article can be reproduced using the data provided by Frýdlová et al. (2026).
