# Supplementary material for: Response of leopard geckos (Eublepharis macularius) towards a multimodal cue simulating a predator
Source: Behav Ecol. 2026 Jun 5;37(4):arag062. doi: 10.1093/beheco/arag062 (PMC13294456; doi:10.1093/beheco/arag062)
Supplement: arag062_Supplementary_Data [file arag062_supplementary_data.pdf]

Response of leopard geckos (*Eublepharis macularius*) towards a multimodal cue simulating a predator

Petra Frýdlová, David Hirschler, Aleksandra Chomik, Eliška Pšeničková, Eva Landová*, Daniel Frynta

This supplementary material summarizes the results of principal component analysis and post hoc tests of the Triple modality test and the Modality test with a live snake.

We employed PCA to explore the behavior of geckos in the Triple modality test. We analysed the duration of the behavior. Three main factors were extracted according to the eigenvalues (>1). PCA revealed a spread of behaviors primarily explained by Factor 1, which accounted for 28.81% of the variance in the data. When expressed as a biplot along with Factor 2, which only explained 15.52% of variance (Figure S1a), active antipredator behaviors (biting, mouth-open threat, high posture, avoiding) clustered together. Factor 1 was characterized by a gradient between defensive and exploratory behavior; high negative loadings for biting, mouth-open threats, and high postures contrasted with tongue-flicking and cases of 'no response'. This suggests a separation between active defensive strategies and exploratory or baseline activities. Factor 2 was further associated with the distinctions between active and passive defensive elements. Factor 3 explained 12.41% of the variance and separated active behavior from no observable response (Figure S1b), which was mostly scored when the animal lay passively and did not do anything.

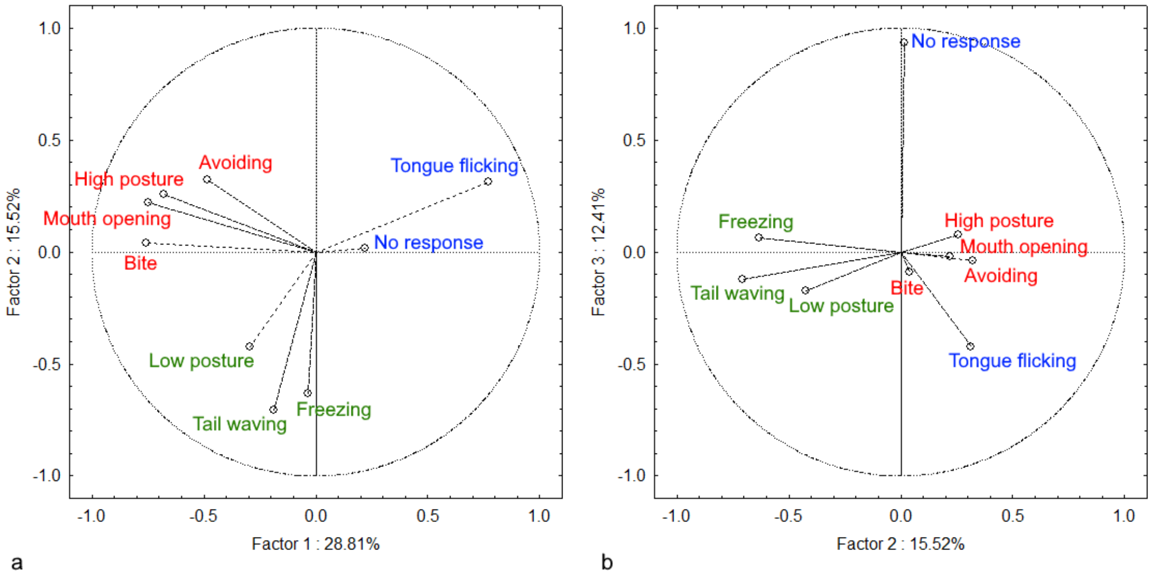

Supplementary figure S1. Behavioral elements of the antipredator reactions in the Triple modality test. Principal component analysis of the duration of behavioral elements observed in the Triple modality test, when the exuvia (shed snakeskin) was present. a) It is visible that Factor 1 splits antipredator behavior (red and green) from exploration (tongue flicking) and ignoring (no response). The second factor separates active antipredator behavior (red) from the passive one (green). b) It is visible that Factor 3 splits all types of active behavior from ignoring the stimulus (no response).

Antipredator behavior occurred in a typical sequence, which we summarize in Figure S2 and is also visible in the video recording (Supplementary Material 2). The reaction to the control treatment is visible in another video recording (Supplementary Material 3). The most common element was tongue flicking, typically followed by a bite or a tail wave. It was also obvious that the detection of a threat is not necessarily preceded by visible tongue flicking, as many reactions began directly with high posture and were followed by a mouth-open threat, bite, or avoidance. Low postures and freezing were rather rare. For visualizing the sequence of behavior, we used the NodeXL Basic plugin, version 1.0.1.448.

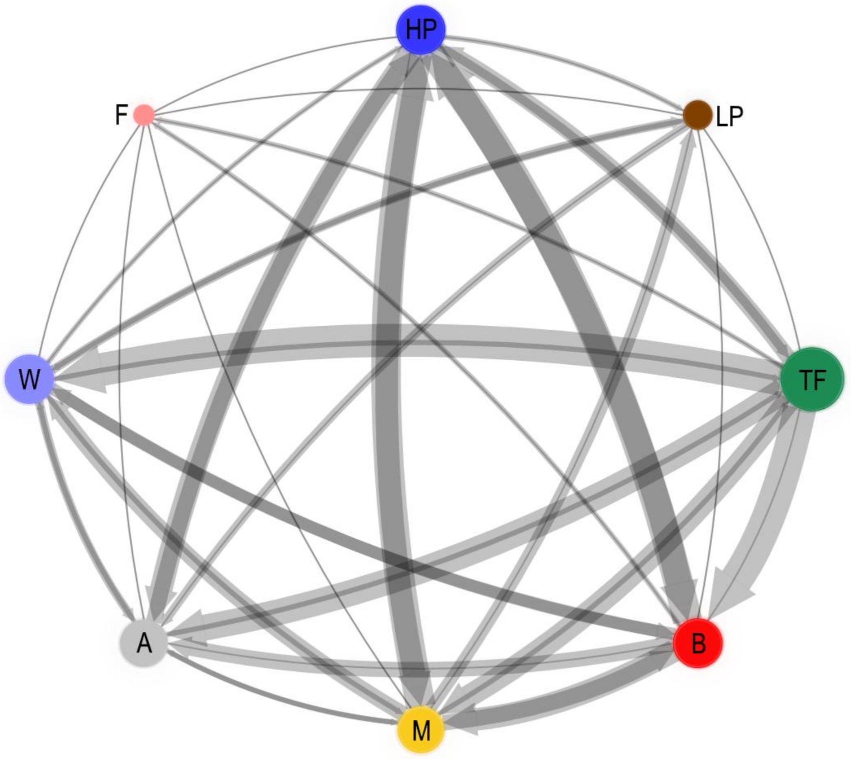

Supplementary figure 2. Behavioral elements of the antipredatory reactions in the Triple modality test. The sequence of behavior during the Triple modality test. The size of the circles and the width of the arrows correspond to the observed quantity of behavior. The direction of the arrows corresponds to the typical sequence of the behavior. It is visible that some behavioral elements are more common than others and that the behavioral elements occur in the typical sequence (e.g., tongue flicks precede tail wave or bite but not the opposite way). Abbreviations: HP (high posture), LP (low posture), TF (tongue flicking), B (bite), M (mouth-opening), A (avoiding), W (tail wave), F (freezing).

Supplementary table S1. Results of the post hoc test for tongue flicking behaviour in the Triple modality test. Statistical differences were analysed using a zero-inflated negative binomial mixed model. Treatment was set as a fixed effect and animal identity as a random factor. P-values were adjusted using the Tukey method. Significant comparisons are in bold.

contrast	contrast	ratio	SE	z-ratio	p-value
T1-T2	Control - Mechanosenzoric	0.79	0.16	-1.19	0.9353
T1-T3	Control - Visual	0.63	0.12	-2.33	0.2755
**T1-T4**	**Control - Chemical**	0.42	0.08	-4.58	**0.0001**
**T1-T5**	**Control - (Visual+Mechanosenzoric)**	0.54	0.10	-3.20	**0.0297**
**T1-T6**	**Control - (Chemical+Mechanosenzoric)**	0.39	0.08	-4.87	**< 0.0001**
**T1-T7**	**Control - (Chemical+Visual)**	0.34	0.06	-5.72	**< 0.0001**
**T1-T8**	**Control - (Chemical+Visual+Mechanosenzoric)**	0.28	0.05	-6.90	**< 0.0001**
T2-T3	Mechanosenzoric - Visual	0.80	0.15	-1.20	0.9321
**T2-T4**	**Mechanosenzoric - Chemical**	0.53	0.10	-3.53	**0.0098**
T2-T5	Mechanosenzoric - (Visual+Mechanosenzoric)	0.69	0.12	-2.08	0.4257
**T2-T6**	**Mechanosenzoric - (Chemical+Mechanosenzoric)**	0.50	0.09	-3.84	**0.0031**
**T2-T7**	**Mechanosenzoric - (Chemical+Visual)**	0.43	0.08	-4.73	**0.0001**
**T2-T8**	**Mechanosenzoric - (Chemical+Visual+Mechanosenzoric)**	0.35	0.06	-5.99	**< 0.0001**
T3-T4	Visual - Chemical	0.67	0.12	-2.22	0.3400
T3-T5	Visual - (Visual+Mechanosenzoric)	0.86	0.16	-0.80	0.9931
T3-T6	Visual - (Chemical+Mechanosenzoric)	0.62	0.12	-2.56	0.1694
**T3-T7**	**Visual - (Chemical+Visual)**	0.54	0.10	-3.40	**0.0154**
**T3-T8**	**Visual - (Chemical+Visual+Mechanosenzoric)**	0.44	0.08	-4.60	**0.0001**
T4-T5	Chemical - (Visual+Mechanosenzoric)	1.29	0.22	1.51	0.8008
T4-T6	Chemical - (Chemical+Mechanosenzoric)	0.93	0.16	-0.41	0.9999
T4-T7	Chemical - (Chemical+Visual)	0.81	0.14	-1.27	0.9104
T4-T8	Chemical - (Chemical+Visual+Mechanosenzoric)	0.66	0.11	-2.55	0.1761
T5-T6	(Visual+Mechanosenzoric) - (Chemical+Mechanosenzoric)	0.72	0.13	-1.88	0.5674
T5-T7	(Visual+Mechanosenzoric) - (Chemical+Visual)	0.63	0.11	-2.78	0.1011
**T5-T8**	**(Visual+Mechanosenzoric) - (Chemical+Visual+Mechanosenzoric)**	0.51	0.08	-4.08	**0.0012**
T6-T7	(Chemical+Mechanosenzoric) - (Chemical+Visual)	0.87	0.15	-0.82	0.9922
T6-T8	(Chemical+Mechanosenzoric) - (Chemical+Visual+Mechanosenzoric)	0.71	0.12	-2.04	0.4542
T7-T8	Chemical+Visual - Chemical+Visual+Mechanosenzoric	0.82	0.13	-1.25	0.9160

Supplementary table S2. Results of the post hoc test for antipredator reaction in the Triple modality test. Post hoc Tukey test for the GLMM model with antipredator reaction as the dependent variable and the type of treatment as a fixed effect. Animal identity was set as a random factor. Significant comparisons are in bold.

contrast	contrast	Odds Ratio	SE	z-ratio	p-value
T1-T2	Control - Mechanosenzoric	1.00	1.50	0.00	1.00
T1-T3	Control - Visual	1.00	1.50	0.00	1.00
T1-T4	Control - Chemical	0.04	0.04	-2.85	0.08
T1-T5	Control - (Visual+Mechanosenzoric)	0.26	0.33	-1.06	0.97
T1-T6	Control - (Chemical+Mechanosenzoric)	0.03	0.04	-2.99	0.06
T1-T7	**Control - (Chemical+Visual)**	0.01	0.02	-3.62	**0.01**
T1-T8	**Control - (Chemical+Visual+Mechanosenzoric)**	0.02	0.03	-3.26	**0.02**
T2-T3	Mechanosenzoric - Visual	1.00	1.50	0.00	1.00
T2-T4	Mechanosenzoric - Chemical	0.04	0.04	-2.85	0.08
T2-T5	Mechanosenzoric - (Visual+Mechanosenzoric)	0.26	0.33	-1.06	0.97
T2-T6	Mechanosenzoric - (Chemical+Mechanosenzoric)	0.03	0.04	-2.99	0.06
T2-T7	**Mechanosenzoric - (Chemical+Visual)**	0.01	0.02	-3.62	**0.01**
T2-T8	**Mechanosenzoric - (Chemical+Visual+Mechanosenzoric)**	0.02	0.03	-3.26	**0.02**
T3-T4	Visual - Chemical	0.04	0.04	-2.85	0.08
T3-T5	Visual - (Visual+Mechanosenzoric)	0.26	0.33	-1.06	0.97
T3-T6	Visual - (Chemical+Mechanosenzoric)	0.03	0.04	-2.99	0.06
T3-T7	**Visual - (Chemical+Visual)**	0.01	0.02	-3.62	**0.01**
T3-T8	**Visual - (Chemical+Visual+Mechanosenzoric)**	0.02	0.03	-3.26	**0.02**
T4-T5	Chemical - (Visual+Mechanosenzoric)	7.55	6.08	2.51	0.19
T4-T6	Chemical - (Chemical+Mechanosenzoric)	0.84	0.49	-0.29	1.00
T4-T7	Chemical - (Chemical+Visual)	0.39	0.22	-1.66	0.72
T4-T8	Chemical - (Chemical+Visual+Mechanosenzoric)	0.61	0.35	-0.86	0.99
T5-T6	(Visual+Mechanosenzoric) - (Chemical+Mechanosenzoric)	0.11	0.09	-2.73	0.11
T5-T7	**(Visual+Mechanosenzoric) - (Chemical+Visual)**	0.05	0.04	-3.66	**0.01**
T5-T8	**(Visual+Mechanosenzoric) - (Chemical+Visual+Mechanosenzoric)**	0.08	0.07	-3.13	**0.04**
T6-T7	(Chemical+Mechanosenzoric) - (Chemical+Visual)	0.46	0.26	-1.38	0.87
T6-T8	(Chemical+Mechanosenzoric) - (Chemical+Visual+Mechanosenzoric)	0.72	0.41	-0.57	1.00
T7-T8	Chemical+Visual - Chemical+Visual+Mechanosenzoric	1.58	0.88	0.83	0.99

Supplementary table S3. Results of the post hoc test for antipredator reaction in the Triple modality test comparing the subset of trials, where the snake exuviae (chemical modality) was presented. Post hoc Tukey test for the GLMM model with antipredator reaction as the dependent variable and the type of treatment as a fixed effect. Animal identity was set as a random factor. Significant comparisons are in bold.

contrast	contrast	Odds Ratio	SE	z-ratio	p-value
T4-T7	Chemical - (Chemical+Visual)	0.36	0.22	-1.70	0.32
T7-T8	Chemical+Visual - Chemical+Visual+Mechanosenzoric	1.64	0.95	0.86	0.83
T6-T7	(Chemical+Mechanosenzoric) - (Chemical+Visual)	0.43	0.26	-1.42	0.49
T4-T8	Chemical - (Chemical+Visual+Mechanosenzoric)	0.59	0.35	-0.89	0.81
T4-T6	Chemical - (Chemical+Mechanosenzoric)	0.83	0.51	-0.30	0.99
T6-T8	(Chemical+Mechanosenzoric) - (Chemical+Visual+Mechanosenzoric)	0.70	0.42	-0.59	0.94

Supplementary table S4. Results of the post hoc test for tongue flicking in the Triple modality test comparing the subset of trials, where the snake exuviae (chemical modality was stimulated) was presented. Statistical differences were analysed using a zero-inflated negative binomial mixed model. Treatment was set as a fixed effect and animal identity as a random factor. P-values were adjusted using the Tukey method. Significant comparisons are in bold.

contrast	contrast	Ratio	SE	z-ratio	p-value
T4-T7	Chemical - (Chemical+Visual)	0.80	0.13	-1.40	0.4987
T7-T8	Chemical+Visual - Chemical+Visual+Mechanosenzoric	0.82	0.13	-1.26	0.5871
T6-T7	(Chemical+Mechanosenzoric) - (Chemical+Visual)	0.86	0.14	-0.95	0.7777
**T4-T8**	**Chemical - (Chemical+Visual+Mechanosenzoric)**	0.66	0.10	-2.70	**0.0348**
T4-T6	Chemical - (Chemical+Mechanosenzoric)	0.94	0.15	-0.39	0.9792
T6-T8	(Chemical+Mechanosenzoric) - (Chemical+Visual+Mechanosenzoric)	0.70	0.11	-2.19	0.1263

Supplementary table S5. Results of the post hoc test for tongue flicking in the Modality test with a live snake. Statistical differences were analysed using a zero-inflated negative binomial mixed model. Treatment was set as a fixed effect and animal identity as a random factor. P-values were adjusted using the Tukey method. Significant comparison is in bold.

contrast	contrast	ratio	SE	z-ratio	p-value
T1-T2	Control - Visual	0.68	0.11	-2.43	0.0722
T1-T3	Control - Chemical	0.68	0.10	-2.52	0.0572
**T1-T4**	**Control - (Chemical+Visual)**	0.50	0.08	-4.44	**0.0001**
T2-T3	Visual - Chemical	0.99	0.15	-0.04	1.0000
T2-T4	Visual - (Chemical+Visual)	0.74	0.11	-2.02	0.1789
T3-T4	Chemical - (Chemical+Visual)	0.74	0.11	-2.04	0.1732

Supplementary table S6. Results of the post hoc test for antipredator reaction in the Modality test with a live snake. Post hoc Tukey test for the LME model with antipredator reaction as the dependent variable and the type of treatment as a fixed effect. Animal identity was set as a random factor. There was no significant comparison.

contrast	contrast	estimate	SE	df	t-ratio	p-value
T1-T2	Control - Visual	-1.15	0.84	164	-1.37	0.5184
T1-T3	Control - Chemical	-2.41	0.97	164	-2.49	0.0648
T1-T4	Control - (Chemical+Visual)	-2.41	0.97	164	-2.49	0.0648
T2-T3	Visual - Chemical	-1.27	0.60	164	-2.10	0.1568
T2-T4	Visual - (Chemical+Visual)	-1.27	0.60	164	-2.10	0.1568
T3-T4	Chemical - (Chemical+Visual)	0.00	0.35	164	0.00	1
